# Knowledge Translation and Implementation Planning to Promote Research Governance in Nongovernment Organizations in the Torres Strait: Descriptive Study

**DOI:** 10.2196/39213

**Published:** 2022-11-01

**Authors:** Sanchia Shibasaki, Felecia Watkin Lui, Lynda Ah Mat

**Affiliations:** 1 ThinkThrough Holland Park Australia; 2 Cairns Institute James Cook University Smithfield Australia

**Keywords:** knowledge translation, implementation planning, research governance, nongovernment organizations, nongovernment organisations, Aboriginal and Torres Strait Islander

## Abstract

**Background:**

Aboriginal and Torres Strait Islander people in Australia have participated in Western research for decades. When done well, research has resulted in significant benefits and positive impacts on society. However, the primary benefactor of this research has and continues to be researchers, with limited or no research knowledge mobilized for uptake and beneficial use by end users, such as individuals and communities. In 2021, the Torres Strait Islanders Research to Policy and Practice Hub (the Hub) at James Cook University designed and implemented several strategies, including a games-based interactive workshop with representatives from nongovernment organizations (NGOs). Feedback suggests the workshop and associated activities were a success.

**Objective:**

We describe knowledge translation (KT) and implementation planning to design and implement strategies to increase awareness and understanding of NGOs in research governance.

**Methods:**

This descriptive study involved representatives from NGOs on Thursday Island in the Torres Strait. We collected data from a literature review and informal discussions. We used several models and frameworks to guide our approach and underpin data collection and analysis.

**Results:**

Designing and implementing strategies to increase awareness and understanding of NGOs in the Torres Strait to govern research involved several key steps: (1) identifying and defining what needed to change and who needed to change, (2) identifying and mapping barriers and facilitators, (3) selecting the most appropriate strategies to support change, (4) implementing activities, and (5) monitoring and evaluating our approach. We developed a program logic to understand and communicate to others how we would implement activities and what resources would be required to support this process. We drew on several evidence-based KT and implementation models and frameworks to do this. First, a KT planning template was used to inform what evidence we wanted to mobilize, to whom, and for what purpose. Based on this step, we recognized we wanted to bring about change with the target audience, and as such, we drew on the previously mentioned implementation planning models and frameworks. We collated the outcomes from these initial steps.

**Conclusions:**

Our KT and implementation practice experience were successful. Encouraging researchers and end users to adopt similar practices requires investment in training and development of KT and implementation practice. This also entails modifying research standards and guidelines to include KT and implementation practice when working with Aboriginal and Torres Strait Islander people and other vulnerable groups, creating incentives for researchers and end users to embed KT and implementation practice in research, and recognizing and rewarding the benefits and impact beyond publication and presentation.

## Introduction

When done well, research has resulted in significant benefits and positive impacts on society [1]. In Australia, Aboriginal and Torres Strait Islander people have participated in Western research for decades [2-4]. However, the primary benefactor of this research has and continues to be researchers, with limited or no research knowledge mobilized for uptake and beneficial use by end users like individuals and communities [5-7]. Our ongoing work with nongovernment organizations (NGOs) in the Torres Strait suggests the following: continued distrust; limited awareness, skills, and experience in research best practices; and little evidence describing the practical application of knowledge translation (KT) and implementation practice by researchers [8,9].

In 2021, the Torres Strait Islanders Research to Policy and Practice Hub (the Hub) at James Cook University designed and implemented several activities, including a games-based interactive workshop with representatives from NGOs in the Torres Strait. Our success is described by a workshop participant: “as a Board member, I can see the importance and why Board members or management committee members or directors should do this training. This is important for us to sit down and get our heads around and understand research, especially when you’re going to be entering into contractual agreements.”

This paper describes KT and implementation planning to design and implement strategies to increase awareness and understanding of NGOs in research governance. Effective KT centers on Aboriginal and Torres Strait Islander communities and their wisdom to achieve maximum research impact through a carefully designed process that minimizes power dynamics and privileges Aboriginal and Torres Strait Islander perspectives. Drawing on other definitions, KT is the reciprocal process of combining experiential wisdom with academic research. It involves a complex series of interactions between knowledge holders, producers, and users to achieve positive and sustainable long-term benefits for Aboriginal and Torres Strait Islander people [10,11]. Implementation is the process of putting to use or integrating new practices within a setting—it is about identifying and defining who needs to change and what individuals need to do differently, understanding and mapping barriers and facilitators, and selecting the most appropriate strategies to support change [12].

## Methods

### Data Collection

**Figure 1:**
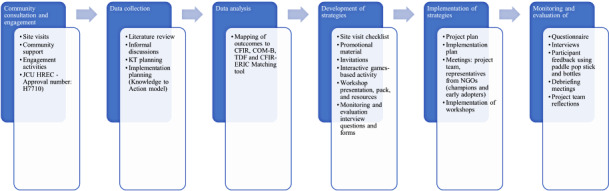
Study approach and methods. CFIR: Consolidated Framework for Implementation Research; CFIR-ERIC: CFIR-Expert Recommendations for Implementing Change; COM-B: Capability, Opportunity, Motivation, Behaviour; JCU HREC: James Cook University Human Research Ethics Committee; KT: knowledge translation; NGO: nongovernment organization; TDF: Theoretical Domains Framework.

Figure 1 shows the methods used in this descriptive study. This paper will focus on the KT and implementation planning activities listed in the boxes titled data collection and analysis in Figure 1.

The study site was Thursday Island in the Torres Strait. The study participants were representatives from NGOs. We adopted the Knowledge to Action process model to guide our KT and implementation planning approach. We collected data from a literature review and informal discussions. The literature review focused on nationally endorsed research guidelines. Representatives from NGOs and project team members participated in informal discussions. We used a KT planning template to identify the evidence to mobilize, who were the intended users of this evidence, what key messages we wanted to share with the specified groups, and what goals we wanted to achieve with each group [13].

### Data Analysis

As shown in Figure 1, we used several frameworks to organize and analyze the data: the Consolidated Framework for Implementation Research (CFIR) [14], the Capability, Opportunity, Motivation, Behaviour (COM-B) [15], the Theoretical Domains Framework (TDF), and the CFIR-ERIC (CFIR-Expert Recommendations for Implementing Change) Implementation Strategy Matching tool, version 1.0 [16].

CFIR described factors to consider in planning for implementation, such as the NGO’s internal and external operating environments. COM-B identified what needed to change for a behavior change intervention to be effective and described barriers to change at the individual level. TDF was used to determine the specific influences on an individual’s behavior. The CFIR-ERIC Matching tool identified the evidence-based strategy that was best suited to address known barriers.

### Ethics Approval

The project obtained ethics approval from the James Cook University Human Research Ethics Committee (approval number: H7710).

## Results

The CFIR factors deemed influential to our approach were (1) outer setting (ongoing demand for research, distrust, limited networking and routine communication between local NGOs, increase demand for NGOs to report on outcomes and impact, limited local research workforce capacity and capability, cultural expectations and requirements about research practice) and (2) inner setting (NGOs are small to medium enterprises [20 to 50 staff], organizations 10 to 20 years of age, governed by a voluntary Aboriginal and Torres Strait Islander board of directors, limited funding and resources for NGOs to do or engage in research, NGOs provide a range of services and may be the only service provider for the region [competing interests and demands], NGOs share similar goals to improve the health and well-being of the local community).

As shown in Table 1, the barriers to research at the individual level were as follows: distrust of researchers and the research process, limited time, limited support to backfill staff to attend training, little or no awareness of research guidelines such as the Keeping Research on Track II, lack of interest, and resistance to change.

Textbox 1 displays the types of intervention strategies that can be used to address these barriers.

**Figure 2:**
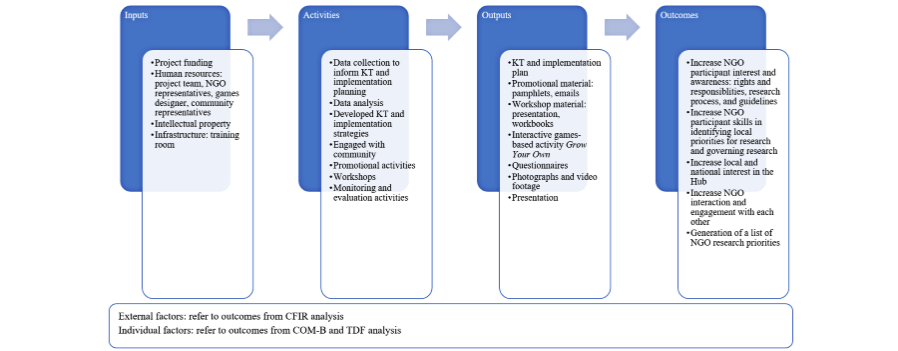
Program logic of study inputs, activities, outputs, and outcomes. CFIR: Consolidated Framework for Implementation Research; COM-B: Capability, Opportunity, Motivation, Behaviour; KT: knowledge translation; NGO: nongovernment organization; TDF: Theoretical Domains Framework.

The program logic in Figure 2 presents our approach to implementing the strategies above.

**Table 1:** Mapping of identified barriers to implementation strategies to implement a set of research guidelines in nongovernment organizations in the Torres Strait.

Barriers, COM-B^a^ domain, and TDF^b^ construct				Implementation strategy characteristics
**Limited or no awareness of research guidelines such as the Keeping Research on Track II**				
	**Capability**			
		Knowledge	Deliver educational workshops Dynamic training Develop and distribute educational resources Tailor approaches to the local context and practice	
**Distrust of researchers and the research process**				
	**Motivation**			
		Emotion: fear, anxiety, and stress	Capture and share local knowledge	
**Lack of familiarity with facilitators**				
	**Motivation**			
		Emotion: fear, anxiety, and stress	Capture and share local knowledge Regular visits to organizations Facilitator-supported activities Identify and co-opt champions	
**Limited time**				
	**Opportunity**			
		Environmental stressors	Invite board members and senior executives	
		Resources	Host events in the local community Reduce participant costs through, for example, free events with catered meals	
**Limited to no support to backfill staff attending training**				
	**Opportunity**			
		Organization culture and climate	Invite board members and senior executives Identify and co-opt champions	
**Resistance to change**				
	**Motivation**			
		Optimism	Identify and co-opt support from local opinion leaders	
		Intentions	Assess readiness Identify environmental and individual barriers and facilitators	
		Goals	Collate outcomes from stakeholder meetings, discussions, and feedback (informal needs assessment)	
		Beliefs about consequences	Same as above	
**Lack of interest**				
	**Capability**			
		Knowledge	Capture and share local knowledge	
	**Opportunity**			
		Social influences	Identify early adopters Identify and co-opt support from local opinion leaders	
	**Motivation**			
		Optimism	Identify and co-opt champions	
		Beliefs about consequences: outcome expectancies	Same as above	

^a^CFIR: Consolidated Framework for Implementation Research.

^b^COM-B: Capability, Opportunity, Motivation, Behaviour.

**Intervention strategies to address barriers.**

- Needs assessment about what needs to change and readiness to change
  Assess readiness
  Identify environmental and individual barriers and facilitators
  Collate outcomes from stakeholder meetings, discussions, and feedback
- Recognizing and embedding environmental barriers and enablers into approaches
  Tailor approaches to the local context and practice
  Capture and share local knowledge
  Invite board members and senior executives
  Host events in the local community
  Reduce participant costs through free events with catered meals
- Developing and implementing strategies based on target audience needs
  Develop and distribute educational resources
  Deliver educational workshops
  Dynamic training
  Facilitator-supported activities
- Gaining target audience and community trust
  Regular visits to organizations
  Identify and co-opt champions, local opinion leaders, and early adopters

## Discussion

### Principal Results

Our approach to designing and implementing strategies to increase awareness and understanding of NGOs in the Torres Strait to govern research involved several steps: (1) identifying and defining what needed to change and who needed to change, (2) identifying and mapping barriers and facilitators, (3) selecting the most appropriate strategies to support change, (4) developing and implementing activities, and (5) monitoring and evaluating our approach.

A KT planning template was used to inform what evidence we wanted to mobilize, to whom, and for what purpose. Based on this step, we recognized we wanted to initiate change with the target audience. We drew on several evidence-based KT and implementation models and frameworks to do this. We drew on the previously mentioned implementation planning models and frameworks. We collated outcomes from these initial steps and developed a program logic to understand how we would implement the strategies and what resources we required to support this process.

The approach we took in this study is not new [14,17,18]. However, there is limited but growing evidence describing the successful use and application of KT and implementation planning practices in NGOs in Aboriginal and Torres Strait Islander communities in Australia [19-21]. By supporting and strengthening these practices, we ensure evidence is mobilized effectively from research to end users. We also enhance end-user capacity and capability to draw on evidence to inform the design and implementation of programs and services in their communities for local benefit and impact. Finally, we demonstrate a systematic approach to inform the decision making of funding authorities and policy makers.

### Strengths and Limitations

Strengths included having Torres Strait Islander researchers and project team members lead and implement the research, a strong level of trust and engagement between researchers and NGOs, and the presence of team capability in KT and implementation planning. The limitations of the project relate to the sample size and study site (all NGOs were in the same remote community). As such, the findings from this project are not generalizable to the broader NGO audience. Timing and time frames were also a limitation. The project timeline was 6 months during COVID-19 restrictions when travel restrictions were in place. The project team could not travel to other parts of Australia to collect data. Furthermore, there were limited project resources to fund an expansion of the project.

### Conclusion

Based on our individual and collective experiences, we know programs, services, and practices are designed and implemented from what we think we know and expect. We have participated in various meetings, forums, and workshops that provided opportunities for participants to catch up and network but were unsuccessful in initiating and sustaining change. We have written journal publications and presented at conferences to enhance our track record but did little to improve investment in local communities. We wanted to disrupt this status quo and embark on an approach to increase awareness and understanding and initiate behavior change. Our KT and implementation practice experience were successful. Encouraging researchers and end users to adopt similar practices will require the following: (1) investment in training and development on KT and implementation practice, (2) modifying research standards and guidelines to include KT and implementation practice when working with Aboriginal and Torres Strait Islander people and other vulnerable groups, (3) creating incentives for researchers and end users to embed KT and implementation practice in research, and (4) recognizing and rewarding benefits and impact beyond the publication and presentation.

## Abbreviations

CFIR: Consolidated Framework for Implementation Research
CFIR-ERIC: CFIR-Expert Recommendations for Implementing Change
COM-B: Capability, Opportunity, Motivation, Behaviour
KT: knowledge translation
NGO: nongovernment organization
TDF: Theoretical Domains Framework

## Data Availability

This paper includes all data generated or analyzed in this study.
